# Scientific Items

**Published:** 1887-09

**Authors:** 


### SCIENTIFIC ITEMS.

**Military Dogs.**—Among the thousand and one inventions, appliances, and wonderful uses of men and beasts which German genius has devised to defeat France in case General Boulanger's successor becomes unpleasant, the dog plays a significant role, employed, as he is, as messenger and sentinel. Experiments have proved highly satisfactory. The dog maneuver of the Hunter battalion was decidedly the most interesting of the recent campaign. Several regiments have been furnished with the German shepherd dogs, known for their wisdom the world over. Each one is attached, so to speak, to the person of a soldier, in whom the dog soon recognizes his master, and who conducts his training. While doing duty, the dog is kept with the sentinel, and easily learns the requirements of his post. A few of the experiments performed before Colonel von der Goltz Pacha, who represented the Sultan at the ninetieth birth day of the Emperor, and has since remained to witness the reviews, were surprising. A soldier, taking the dog from the sentinel, marched off on a reconnoitering expedition. After writing his observations, and placing them in a cask about the neck of the brute, the latter was told to return to his master, which he did in an astonishingly short time. One dog employed in this service arrived at his post ten minutes before a mounted Uhlan charged with the same instructions, though the latter rode at desperate speed. But even more than this was accomplished. With a message tied about the neck, as in the former case, the dog was told to seek a distant sentinel and bring a return answer. This he did with great speed, carrying his message directly to his master without fail. It is little wonder that Pacha Goltz was surprized at the success of the experiments given in his honor. And they are truly wonderful for the present, though bidding fair to become a common-place institution in that great machine, the German army. The consequences and possibilities of the shepherd dog service are apparent to all who know anything of military science, and make their citation superfluous. One thing is certain, that a future war between Germany and any of its neighbors will not be conducted without its dog regiment, which, though not employed in concerted action, will perform service more valuable than the cats of ancient Egypt.—*Correspondence Tribune.*

**A harrow-shaped** flock of wild geese, the Waterbury (Conn.) *American* says, went northward over the city recently. They seemed to attend sharply to the business of traveling until they spied one of the numerous kites the boys in the northern portion of the city were flying. This kite was uncommonly high in the air, and the geese objected to it. At least they circled about it two or three times, and then four of their number, delegated for the purpose, attacked the kite and tore it into shreds, and went on their way.—*Scientific American.*

**An observer** down South says an alligator's throat is an animated sewer. Everything which lodges in his open mouth goes down. He is a lazy dog, and instead of hunting for something to eat he lets his victuals hunt for him. That is, he lies with his great mouth open, apparently dead, like the 'possum. Soon a bug crawls into it, then a fly, then several gnats, and a colony of mosquitoes. The alligator doesn't close his mouth yet. He is waiting for a whole drove of things. He does his eating by wholesale. A little later a lizard will cool himself under the shade of the upper jaw. Then a few frogs will hop up to catch the mosquitoes. Then more mosquitoes and gnats will light on the frogs. Finally a whole village of insects and reptiles settle down for an afternoon picnic. Then all at once there is an earthquake. The big jaw falls, the alligator blinks one eye, gulps down the entire menagerie, and opens his great front door again for more visitors.—*Scientific American.*

**A New Light for Instantaneous Photographs.**—At a recent meeting of the Berlin Physical Society, Prof. C. W. Vogel communicated the most recent discovery in connection with instantaneous photography, by which it is now possible to obtain instantaneous photographs not only at night, but also in the darkest places. Messrs. Gœdicke and Miethe have prepared a mixture of pulverized magnesium, chloride of potash, and sulphide of antimony, which when ignited produces an explosive, lightning-like illumination of such intensity that by means of it an instantaneous photograph can be taken. The speaker then gave a demonstration of the discovery by taking photographs of several persons present. He used the artificial light, of which each flash lasted one-fortieth of a second, and in a few minutes produced a picture during the meeting. The powders, as prepared by the discoverers, cost only a few pfennigs each, and will hence readily come into general use.—*Ib.*

**Do not Eat Raw Eggs.**—In the *Monatschrift zum Schutze der Vogewelt*, Professor Liebe adduces reliable data in answer to the question whether living worms are to be found in hens' eggs. A short time previously his sister had found a round, thread-like worm, the length of a little finger, in the white of an egg. It moved itself in a very lively manner. She at once took the white of the egg to a druggist, who put the worm in alcohol. Professor Mobius, of Kiel, decided that the specimen was an example of the thread worm of fowls—*Heteratis inflexa*—often found in the small intestine of the domestic hen. Only a few instances of the existence of the same in the white of the egg have been recorded.—*Allgemeine Medicinsiche Central-Zeitung.*

**A Lense** which magnifies, and yet is perfectly flat on both sides, is a scientific novelty. It is made at Jena, by the manufacturer of Professor Abbe's new optical glass. The lens consists of a single disk, whose density varies so that its refractive power decreases regularly from the surface inward.

